# Dynamic ulnar nerve compression at the elbow in a collegiate baseball player due to aberrant branch of the brachial artery

**DOI:** 10.1016/j.xrrt.2021.04.005

**Published:** 2021-04-28

**Authors:** J. Weston Robison, Jeremy T. Royal, Gary M. Lourie

**Affiliations:** a Wellstar Atlanta Medical Center, Atlanta, GA, USA; b Northside Hospital, Atlanta, GA, USA; c The Hand and Upper Extremity Center of Georgia, Atlanta, GA, USA

**Keywords:** Athletes, Baseball, Elbow, Nerve compression syndromes, Ulnar neuropathies

Dynamic nerve compressive neuropathies at the elbow are rare and require a high level of suspicion in the diagnostic process. Even published literature on the topic is relatively limited, but most cases share an exercise-induced neuropathic presentation which completely resolves at rest.^1^, ^2^, ^3^^,^^6^^,^^7^^,^^9^ To date, there have been no cases of compressive ulnar neuropathy at the elbow owing to an aberrant artery crossing and compressing the ulnar nerve, resulting in activity-induced nerve symptoms that are completely asymptomatic and fully resolved at rest. We present a case of a dynamic elbow compressive ulnar neuropathy, in a high-level collegiate pitcher, that resulted from compression by an aberrant artery in the arm.

## Case report

An elite, collegiate, right-handed, baseball pitcher presented with a one-year history of medial elbow symptoms 3-4 centimeters proximal to the medial epicondyle, localized specifically to the intermuscular septum, presenting only after 2-3 innings of pitching and completely resolving at rest. On direct questioning, he described his symptoms as pain and soreness to touch over a specific area 3-4 centimeters proximal to the medial epicondyle associated with numbness in the ulnar 2 digits, and with continued pitching, he experienced a loss in command and control and a drop in velocity. Initial examination at rest revealed a nontender, stable elbow with a full range of motion. Provocative testing for ulnar collateral ligament stability was within normal limits. The ulnar nerve was stable and did not subluxate with elbow flexion. With a normal history and examination at rest and with a high index of suspicion of a dynamic nature to his presentation, possibly because of an anomalous muscle or aberrant structure compressing vital structures, he was instructed before the next visit to throw for 2-3 innings until symptoms were present. At that office visit, he reported that the pain stemmed from an area approximately 3 cm proximal to the medial epicondyle directly over the intermuscular septum. Palpation of this area caused numbness in the ulnar 2 digits with a positive Tinel’s sign at this location only and not over the cubital tunnel proper.

### Imaging and nerve studies

**Figure 1:**
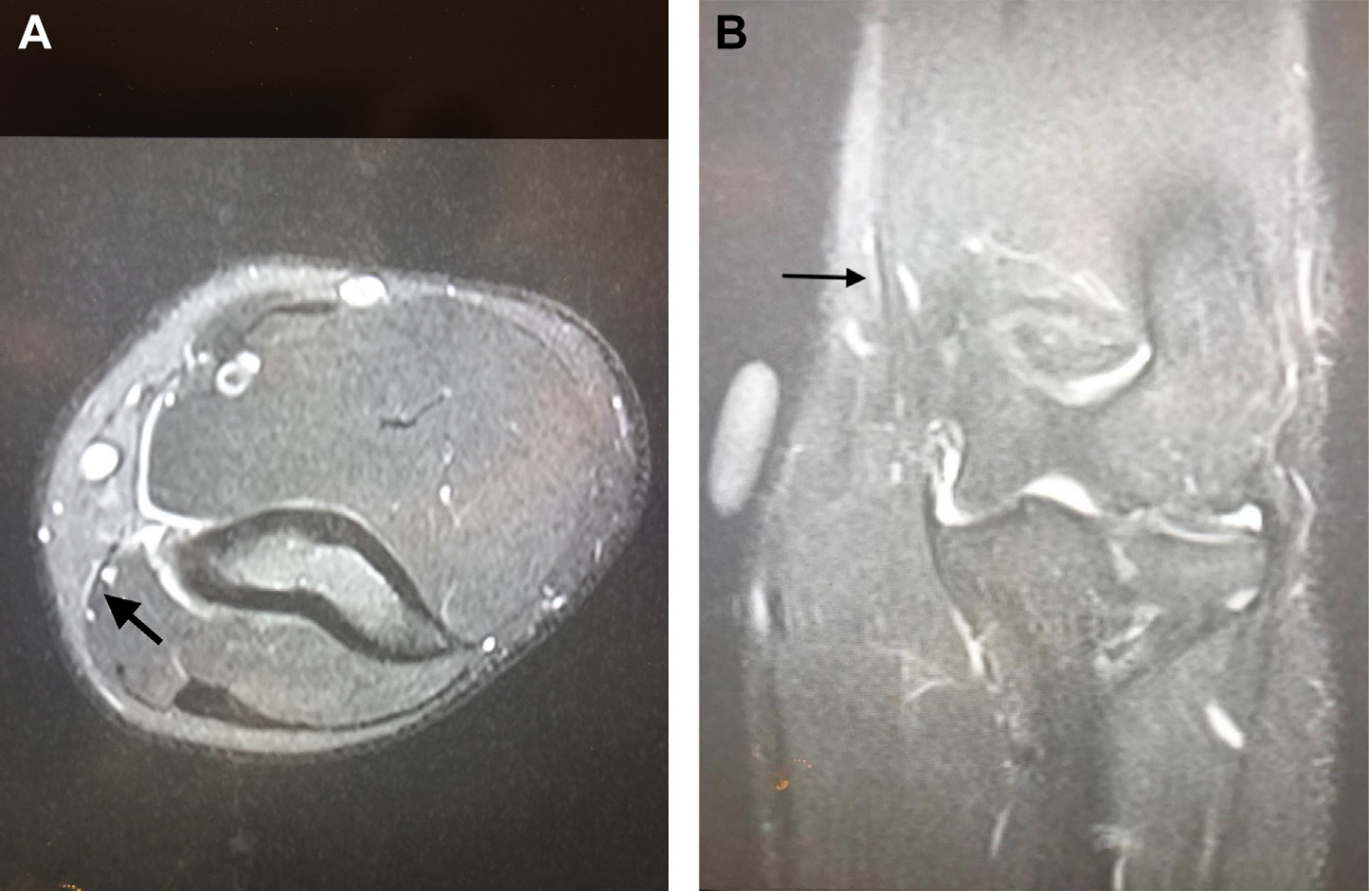
Magnetic resonance imaging scan of the left elbow demonstrates ulnar nerve abutment with a branch of the superior ulnar collateral artery. (**A**) Axial; (**B**) Coronal.

**Figure 2:**
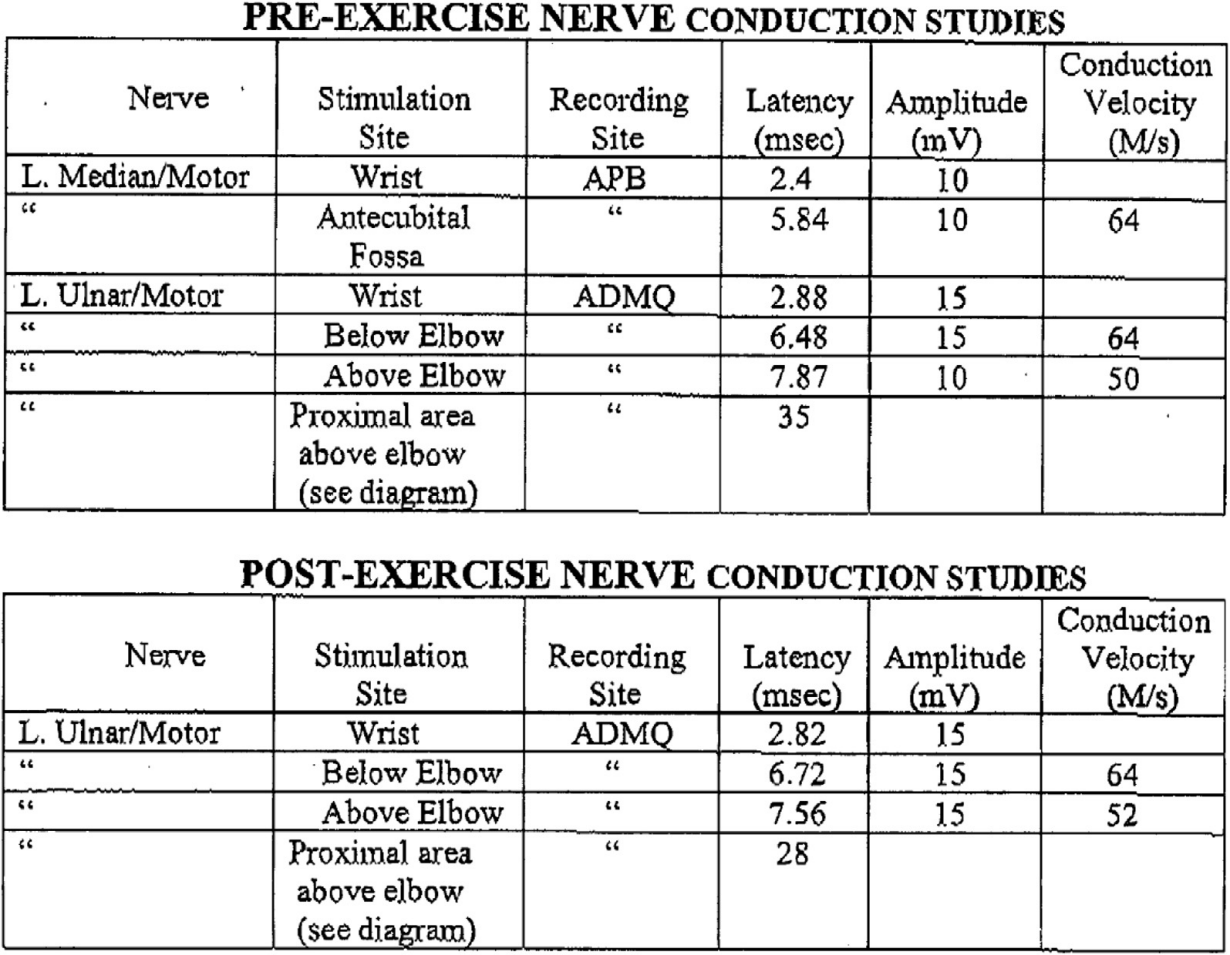
Dynamic nerve conduction studies.

With a high index of suspicion for a dynamic ulnar nerve entrapment, he underwent dynamic pre-exercise and postexercise nerve studies coupled with an inching technique to better define the exact location of compression. The inching technique is a nerve conduction study technique wherein numerous stimulus applications are applied over many short segments of a nerve in attempts to identify an area of focal slowing. In addition, magnetic resonance imaging (MRI) was performed with guided instructions to place a vitamin E capsule corresponding to the area of maximal tenderness (Fig. 1). Nerve conduction studies were conducted at rest and again immediately after he pitched, at which time he became symptomatic. Postexercise dynamic nerve studies with inching technique revealed a 20% reduction (35 msec before pitching, 28 msec after pitching) in latencies in the ulnar nerve 3 cm proximal to the medial epicondyle, which corresponded to the area of his pain (Fig. 2). The MRI demonstrated signal changes in the ulnar nerve approximately 3 cm proximal to the medial epicondyle. A diagnosis of dynamic left ulnar nerve neuropathy was made, and studies pointed to a localized area of compression of the nerve 3 cm proximal to the epicondyle that was responsible for the symptoms that worsened with activity and resolved at rest.

### Surgical findings

**Figure 3:**
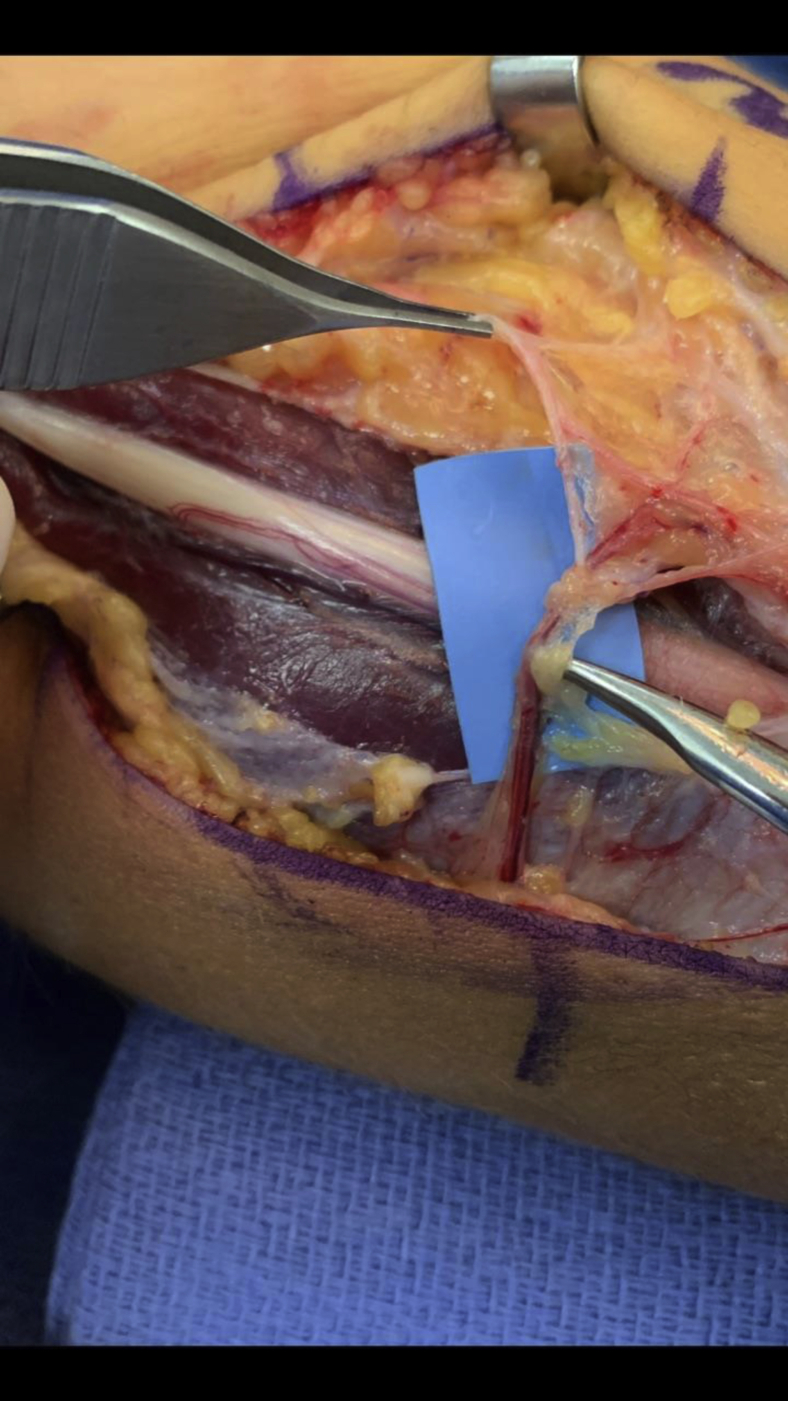
Intraoperative photograph of aberrant transverse branch off of the superior ulnar collateral artery compressing the ulnar nerve.

With the knowledge gained from the studies and failed improvement with rest and physical therapy, he underwent exploration of the ulnar nerve in the distal arm and cubital tunnel. Intraoperative dissection revealed a prominent aberrant arterial branch arising from the superior ulnar collateral artery 3 cm proximal to the medial epicondyle. This branch crossed the intermuscular septum compressing the ulnar nerve and corresponded exactly to the area the preoperative studies confirmed as a possible pathologic source of the symptoms (Fig. 3). The nerve studies, specifically the inching technique, showed a localized drop in velocity at this area, while the MRI revealed a signal change in the nerve as well. This aberrant branch was ligated to release compression across the nerve at this level, and in addition, the nerve was transposed anteriorly to avoid later compression in a high-level pitcher.

### Outcome

His postoperative course was unremarkable. Four weeks of immobilization was followed by range-of-motion exercises, and at 3 months postoperatively, an interval throwing program was begun. At 6 months, he was pitching without symptoms with complete recovery of command, control, velocity, and endurance. He is now at 18 months and continues to pitch without any sequelae. Patient consent was obtained for the case report.

## Discussion

**Figure 4:**
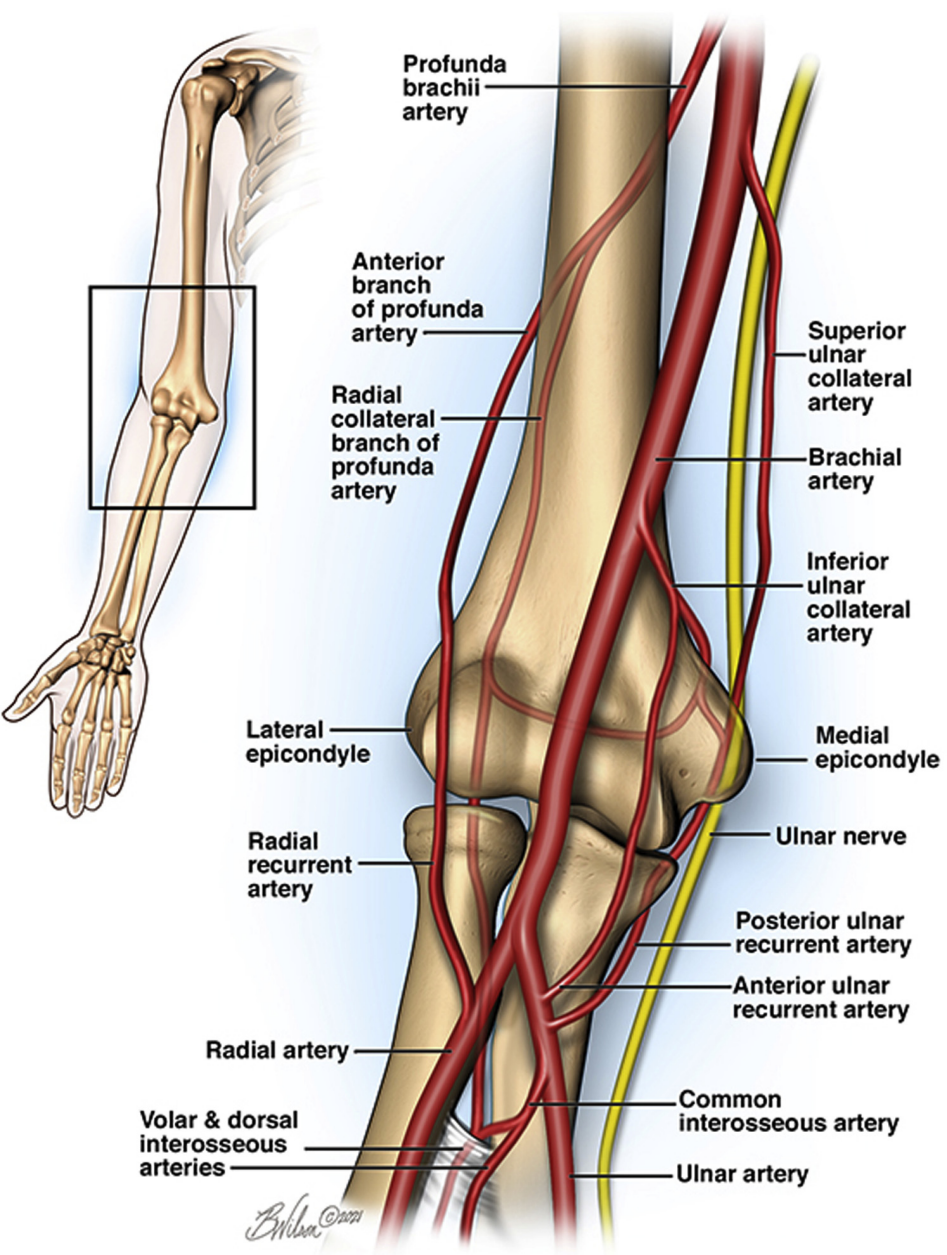
Superior ulnar collateral artery at the elbow. Illustration complements of Brian Wilson.

Dynamic ulnar nerve compressive neuropathies at the elbow are relatively rare, with the senior author recently publishing such a series.^9^ In this select group, high-level pitchers were treated for a dynamic presentation of nerve entrapment around the elbow. While the ulnar, radial, and musculocutaneous nerves were all treated, all shared a common etiology of either an anomalous or hypertrophied muscle causing exercise-induced nerve compression, relieved with surgical decompression of the particular source of entrapment. A recent case report of dynamic compression of the ulnar nerve at the level of Guyon’s canal, due to an anomalous deep branch of the ulnar artery, has been described.^5^ However, to our knowledge, this is the first reported case of a dynamic compressive ulnar neuropathy resulting from an aberrant vessel in the arm. The main extrinsic vascular supply to the ulnar nerve about the cubital tunnel has been previously described in cadaver studies,^4^^,^^8^^,^^10^ including the superior ulnar collateral artery, the inferior ulnar collateral artery, and the posterior ulnar recurrent artery (Fig. 4). The incidence of aberrant vessels about the ulnar collateral arterial system is not known. They may only become clinically significant in a dynamic fashion, as seen in athletes with repetitive use of the extremity, such as in high-level baseball pitchers or tennis players.

## Conclusions

In the described case, the dynamic nature and location of his pain directly correlated with the inching technique nerve conduction study and MRI findings; intraoperative findings confirmed the presence of an aberrant vessel at this location. These findings reiterate the importance of a thorough history and physical examination. This high-level collegiate pitcher exemplifies a unique case of dynamic nerve compression. In cases of suspected dynamic nerve compression in a high-level athlete, additional diagnostic and imaging modalities, such as dynamic nerve studies with inching technique and dynamic MRI, are warranted if initial history and physical examination do not reveal an etiology. The absence of findings at rest highlights the importance of a high index of suspicion for this rare, but potentially incapacitating diagnosis that with an astute awareness, can lead to a successful surgical outcome.

## Disclaimers

Funding: No funding was disclosed by the author(s).

Conflicts of interest: The authors, their immediate families, and any research foundations with which they are affiliated have not received any financial payments or other benefits from any commercial entity related to the subject of this article.

Patient consent: Obtained.
